# Coping with burnout and the impact of the COVID-19 pandemic on workers’ mental health: A systematic review

**DOI:** 10.3389/fpsyt.2023.1139260

**Published:** 2023-03-16

**Authors:** Maria Francesca Rossi, Maria Rosaria Gualano, Nicola Magnavita, Umberto Moscato, Paolo Emilio Santoro, Ivan Borrelli

**Affiliations:** ^1^Section of Occupational Health, Department of Life Sciences and Public Health, Università Cattolica del Sacro Cuore, Rome, Italy; ^2^School of Medicine, UniCamillus-Saint Camillus International University of Health Sciences, Rome, Italy; ^3^Leadership in Medicine Research Center, Università Cattolica del Sacro Cuore, Rome, Italy; ^4^Center for Global Health Research and Studies, Università Cattolica del Sacro Cuore, Rome, Italy; ^5^Department of Woman and Child Health and Public Health, Fondazione Policlinico Universitario Agostino Gemelli IRCCS, Rome, Italy

**Keywords:** burnout, coping, COVID-19, occupational health, workers

## Abstract

**Introduction:**

The COVID-19 pandemic had a negative impact on the psychological wellbeing of workers worldwide. Certain coping styles may increase burnout risk. To investigate the relationship between burnout and coping styles, a systematic review was performed.

**Methods:**

Following the PRISMA statements, three databases were screened up until October 2022, including research articles written in English language and investigating the relationship between burnout and coping strategies in workers. The quality of articles was assessed by the Newcastle-Ottawa Scale.

**Results:**

The initial search resulted in 3,413 records, 15 of which were included in this review. Most studies were performed on healthcare workers (*n* = 13, 86.6%) and included a majority of female workers (*n* = 13, 86.7%). The most used burnout assessment questionnaire was the Maslach Burnout Inventory (*n* = 8, 53.3%), and the most used coping assessment tool was the Brief-COPE (*n* = 6, 40.0%). Task-related coping was a protective factor for burnout in all four studies investigating its correlation with burnout dimensions. Two of the four studies investigating emotion-oriented coping found that it was protective while the other two found that it was predictive of burnout. All five studies investigating avoidance-oriented coping and burnout dimensions found that this coping style was predictive of burnout.

**Discussion:**

Task-oriented and adaptive coping were protective for burnout, avoidance-oriented, and maladaptive coping were predictive factors of burnout. Mixed results were highlighted concerning emotion-oriented coping, suggesting that different outcomes of this coping style may depend on gender, with women relying more on it than men. In conclusion, further research is needed to investigate the effect of coping styles in individuals, and how these correlates with their unique characteristics. Training workers about appropriate coping styles to adopt may be essential to enact prevention strategies to reduce burnout incidence in workers.

## 1. Introduction

The COVID-19 Pandemic has been ongoing since 2020, with a significant negative impact on workers’ health worldwide, not just limited to physical wellbeing, but also affecting psychological wellbeing (1–4). COVID-19 has had an impact on occupational health both directly and indirectly. The direct impact was having to manage the contagion risk in the workplace and returning to work post COVID-19 (4), as well as the emerging problems related to long COVID-19 syndrome that are currently still being evaluated (5). The indirect effect of COVID-19 on the workplace has been on the organizational measures adopted to reduce its impact, remote working being the primary solution for many companies to reduce contagion risk; remote working has been itself an important source of psychological distress for workers worldwide (6).

### 1.1. Burnout definition

Burnout is defined as a psychological condition characterized by tiredness, cynicism, and ineffectiveness at work, which affects how employees perceive themselves and others at work (7). Although the condition of the worker suffering from burnout is quite widespread and easily recognizable, there is no agreement among researchers about the criteria to be used for risk assessment (8). In research on burnout both unidimensional and multidimensional models are used. The unidimensional models only have one dimension, exhaustion. Some unidimensional models make a distinction between psychological and physical exhaustion, although exhaustion as a single factor is used to perform burnout measurements (9). Exhaustion is defined as the fatigue experienced by workers, caused by the chronic depletion of their emotional resources, leading to the emotional withdrawal of the employee from their job, worsening their health status, and work performances (10). The multidimensional models of burnout have three primary components: emotional exhaustion, depersonalization, and reduced personal accomplishment (11). Depersonalization is defined as the progressive dehumanization of the workers’ clients, leading to the perception that the clients deserve the troubles they have (10). This aspect of burnout is correlated to the progressive emotional exhaustion (as defined above) of the worker, which leads to reduced empathy. Both depersonalization and emotional exhaustion positively correlate with burnout, meaning that high scores of these components translate into a high burnout level (11). Personal accomplishment is defined as the satisfaction perceived by the workers in regards to their job performances (10). This dimension negatively correlates with burnout, with high personal accomplishment meaning lower burnout levels (11).

### 1.2. Burnout assessment tools

Many tools have been developed to measure burnout objectively in workers: the first questionnaire was developed in 1981 by Maslach et al. (10), but many other instruments are now available and validated. Among the most popular, there are the Copenhagen Burnout Inventory, the Oldenburg Burnout Inventory, the Bergen Burnout Inventory, the Professional Quality of Life, the Burnout Assessment Tool (9, 12–14).

### 1.3. Burnout during the COVID-19 pandemic

The COVID-19 pandemic has stimulated a large number of studies on workers’ mental health, and many of these have reported a high prevalence of burnout (15, 16). Longitudinal studies, with the comparison between data collected before the pandemic and subsequent observations, are currently few (17). An umbrella review of studies conducted during pandemics has shown that during such outbreaks healthcare workers suffer from high burnout rates, but the prevalence rates observed are like those recorded outside the pandemic in healthcare sectors with high stress (18). Repeated cross-sectional studies measuring burnout levels at the start of the pandemic have observed a reduction in prevalence over time (19, 20), especially in cases in which interventions to support workers have been adopted (21). As burnout became more prevalent during the COVID-19 pandemic, a review was performed to assess which behaviors in healthcare workers resulted in the lowest burnout scores: social and emotional support, physical activity, physical self-care, and emotional and physical distancing from work were the most effective coping strategies in healthcare personnel (22).

### 1.4. Coping definition and coping styles

The extent of the effects that appear in workers could be related to the type of response they implement against stress factors. Coping is defined as an “organizational construct” referring to the multitude of actions and behaviors that a person can use in order to deal with psychological distress (stress, anxiety, and burnout) (23). Classifying coping mechanisms has proved to be a difficult task, because people can react to stressors very differently, and there is no limited set of behaviors or beliefs that can be used to cope with stress (23). Coping is described by different styles, and can be either problem-focused, emotion-focused or avoidance-focused. Problem-focused coping means acting on the source of the psychological distress using an active approach; emotion-focused coping means managing the emotional response to the psychological stressor (24); avoidance-focused coping, can occur when the individual tries to ignore the stressor through social support or through distracting activities (25). If the coping strategies adopted by the individual are ineffective in attenuating work-related stress, the psychological distress can progressively led to burnout (26). For example, problem-focused coping strategies in situations where the problem cannot be solved or in chronic situations can lead to ineffective coping (27), on the other hand, emotion-focused coping strategies that lead the individual to dethatch themselves from the issue can be just as ineffective (28).

### 1.5. Coping assessment tools

Due to the heterogeneity of coping strategies and styles, many different tools have been developed and validated to assess coping in workers (according to our review of the literature, the most relevant are the Cybernetic Coping Scale, the Coping Orientation to Problems Experienced or COPE Inventory, the Brief-COPE, the Perceived Ability to Cope with Trauma scale, the Simplified Coping Style questionnaire, the Trait Coping Style Questionnaire, the Brief Resilient Coping Scale, the Coping Inventory for Stressful Situations) (25, 29–34).

The individual’s ability to avoid developing burnout is related not only to the work environment and occupational stressors, but also to the worker’s coping skills. During the COVID-19 pandemic burnout levels and its incidence have been rising in workers worldwide, therefore many coping strategies have been adopted by employees to reduce the negative psychological impact of the pandemic.

### 1.6. Aim of the review

Since the relationship between burnout and coping strategies is still being investigated in scientific literature, and given the higher burnout prevalence in employees during the current pandemic, the aim of this systematic review was to assess the correlation between burnout and coping strategies during the COVID-19 pandemic in workers.

## 2. Materials and methods

The systematic review was performed following the Preferred Reporting Items for Systematic Reviews and Meta-Analyses (PRISMA) statements (35). Three databases were selected due to their relevance in the medical and biomedical fields: PubMed, ISI Web of Knowledge, and Scopus (36).

A query was developed following the PICO model, establishing the Population (P) as workers, the Intervention (I) as measuring coping and/or coping strategies in workers, and the Outcome (O) as burnout. Comparison (C) was not applicable due to the aim of the performed review. The query used to perform the bibliographic search was comprised as follows: burnout AND coping AND workers.

The research was restricted to articles investigating coping mechanism adopted by workers suffering from – or trying to prevent – burnout, during the COVID-19 pandemic, published from the onset of the pandemic (December 2019) up to October 2022, when the last search was performed; manuscripts in English were included in the review. Studies that did not use validated questionnaires or used semi-structured interviews (mixed method research) to measure coping or burnout, and studies that were not performed on workers were excluded. Non-research articles (wrong publication type, i.e.,: commentaries, letters, and editorials), secondary studies (review and meta-analyses), and manuscripts written in language other than English were also excluded. We also excluded studies from time periods prior to the pandemic but published within the past 2 years.

After retrieving the articles from all the selected databases, duplicate removal and the initial screening by title and abstract was performed through the website Rayyan (37), which allowed for articles to be screened by the three researchers independently, following triple blind methodology, in order to reduce selection bias.

A quality assessment was performed for the included studies using the Newcastle-Ottawa Scale (NOS) (38).

Data was extracted and reported in an Excel sheet and results were presented quantitatively.

## 3. Results

**FIGURE 1:**
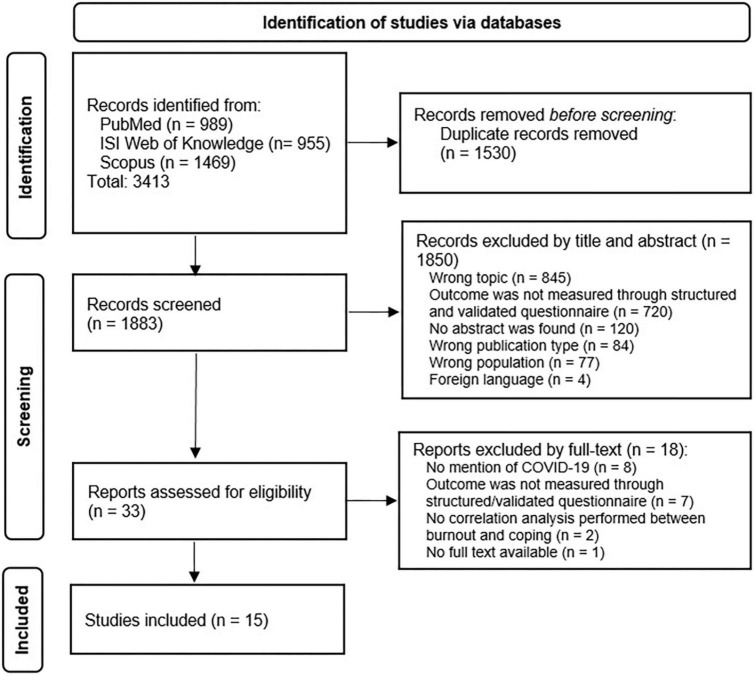
PRISMA flowchart.

The initial systematic search resulted in 3,413 records found across the three databases (PubMed, ISI Web of Knowledge, and Scopus). After removing 1,530 duplicates, 1,883 manuscripts resulted eligible for screening. The screening by title and abstract resulted in a total of 1,850 excluded articles (see Figure 1 for exclusion motivations). The remaining 33 articles were screened by full text; all the articles were successfully retrieved. A total of 18 articles were excluded based on full text (see Figure 1 for exclusion motivations), leaving 15 studies to be included in this systematic review (Table 1) (39–53).

**TABLE 1:** Extraction table with main characteristics of population studied.

References	Country	Type of workers	Data gathering period	Mean age of workers (SD)	Sample size	Male workers (%)	Female workers (%)
Lungulescu et al. (39)	Romania	Healthcare workers	1 Nov–15 Dec 2021	30.09 ( ± 3.81)	122	27 (22.1%)	95 (77.87%)
Crescenzo et al. (40)	Italy	Psychologists	Mar-May 2020	40.95 ( ± 8.98)	468	77 (16.5%	391 (83.5%)
Vancappel et al. (41)	France	Healthcare workers	24 Mar – 28 Jun 2020	39.24 ( ± 11.13)	1,010	172 (17.0%)	838 (83.0%)
Michela et al. (42)	Italy	Healthcare workers	Feb-Mar 2021	43.85 ( ± 10.75)	1,009	351 (34.8%)	658 (65.2%)
Di Monte et al. (43)	Italy	Healthcare workers	10 Mar–18 May 2020	55.13 ( ± 11.40)	102	37 (36.3%)	64 (62.7%)
Köse et al. (44)	Turkey	Healthcare workers	Apr-Jun 2020	27.80 ( ± 7.48)	129	18 (13.5%)	111 (86.05%)
Zhang et al. (45)	China	Healthcare workers	1 Mar–8 Mar 2020	median age 33 years (IQR 28,39)	946	276 (29.2%)	670 (70.8%)
Liu et al. (46)	China	Healthcare workers	9 Feb–11 Feb 2020	20–29: 198; 30–39: 406; 40–49: 191; ≥ 50: 85	880	279 (31.7%)	601 (68.3%)
Pang et al. (47)	Sabah	Healthcare workers	1 Dec 2020–30 Apr 2021	134 (89.3%) < 40 years; 16 (10.7%) ≥ 40 years:	150	33 (22.0%)	117 (78.0%)
Fteropoulli et al. (48)	Cyprus	Healthcare workers	25 May–27 Oct 2020	36.86 ( ± 8.93)	1,071	289 (27.0%)	782 (73.0%)
AlJhani et al. (49)	Saudi Arabia	Healthcare workers	Jul–Sep 2020	60.5% were age group 22–35 years	403	97 (24.1%)	306 (75.9%)
Fonseca et al. (50)	Portugal	Healthcare workers	NR	38.54 ( ± 5.37)	111	61 (55.0%)	50 (45.0%)
Finuf et al. (51)	USA	Healthcare workers	22 Sep–31 Oct 2020	44.1 years ( ± 13.1)	44	7 (16.3%)	37 (83.7%)
Sumner et al. (52)	UK and Ireland	Frontline workers	31 Mar–15 May 2020		1,305	196 (13.3%)	1109 (86.7%)
Miller et al. (53)	USA	Healthcare workers	Nov 2020	34 (range 20–63)	200	152 (76.0%)	48 (24.0%)

Out of the fifteen included studies, three were conducted in Italy (20.0%) (40, 42, 43), two in China (13.3%) (45, 46), two in USA (13.3%) (51, 53), one each (6.7%) in the following countries: Cyprus (48), France (41), Portugal (50), Romania (39), Sabah (47), Saudi Arabia (49), Turkey (44), UK and Ireland (52). Thirteen studies were conducted on healthcare workers (86.6%) (39, 41–51, 53), one on frontline workers (6.7%) (52), and one on psychologists (6.7%) (40). Concerning sample size, one study (6.7%) had a sample ≤ 100 (51), six (40.0%) had a sample size between 101 and 200 (39, 43, 44, 47, 50, 53), two (13.3%) had a sample size between 201 and 500 (40, 49), two (13.3%) between 501 and 1,000 (45, 46), and four (26.6%) had a sample size ≥ 1,000 (41, 42, 48, 52). Most included studies (*n* = 13, 86.6%) included a majority of female workers (39–49, 51, 52), and only two studies (13.3%) included a majority of male workers (50, 53).

All studies had a cross-sectional epidemiological design.

The most used burnout assessment questionnaire was the Maslach Burnout Inventory, used in 8 (53.3%) of the 15 included studies (39–46), followed by the Copenhagen Burnout Inventory used in three studies (20.0%) (47–49), the Oldenburg Burnout Inventory was used in two (13.3%) studies (50, 51), and the Bergen Burnout Inventory (52) and Professional Quality of Life questionnaires (53) were used in one (6.7%) study each. Concerning coping assessment tools, the Brief-COPE was used in 6 studies (40.0%) (41, 47–50, 53), the COPE in two (13.3%) (39, 40), the Coping Inventory for Stressful Situations in two (13.3%) (42, 43), the Perceived Ability to Cope with Trauma scale (44), the Simplified Coping Style questionnaire (45), the Trait Coping Style Questionnaire (46), the Brief Resilient Coping Scale (52), and the Cybernetic Coping Scale (51) were used in one study each (6.7%) (Table 2).

**TABLE 2:** Extraction table with main results for each included study; studies are ordered based on burnout assessment questionnaire (most frequently used to least), coping questionnaire (most frequently used to least), and newest to oldest.

References	Burnout questionnaire	Coping questionnaire	Correlation between Burnout and coping	Value	Significance
Lungulescu et al. (39)	Maslach Burnout Inventory	COPE	Burnout and Active approach	−0.51	<0.001
			Burnout and Planning	−0.46	<0.001
			Burnout and Deletion of Concurrent Activities	−0.13	NS
			Burnout and Restraint	0.01	NS
			Burnout and use of Social-instrumental Support	−0.16	NS
			Burnout and use of Social-emotional Support	−0.11	NS
			Burnout and Positive interpretation	−0.47	<0.001
			Burnout and Acceptance	−0.20	<0.05
			Burnout and Religious approach	0.09	NS
			Burnout and Denial	0.34	<0.001
			Burnout and Expressing Emotions	0.35	<0.001
			Burnout and Mental Deactivation	0.35	<0.001
			Burnout and Behavioral Deactivation	0.64	<0.001
			Burnout and Substance Abuse	0.15	NS
Crescenzo et al. (40)	Maslach Burnout Inventory	COPE	Emotional Exhaustion and Avoidance strategies	0.39	<0.01
			Emotional Exhaustion and Trascendental Orientation	0.03	NS
			Emotional Exhaustion and Positive Attitude	−0.32	<0.01
			Emotional Exhaustion and Social Support	0.01	NS
			Emotional Exhaustion and Orientation to the Problem	−0.09	<0.05
			Depersonalization and Avoidance strategies	0.40	<0.01
			Depersonalization and Transcendental Orientation	0.01	NS
			Depersonalization and Positive Attitude	−0.29	<0.01
			Depersonalization and Social Support	−0.13	<0.01
			Depersonalization and Orientation to the Problem	−0.05	NS
			Personal Realization and Avoidance strategies	−0.37	<0.01
			Personal Realization and Transcendental Orientation	−0.01	NS
			Personal Realization and Positive Attitude	0.42	<0.01
			Personal Realization and Social Support	0.14	<0.01
			Personal Realization and Orientation to the Problem	0.17	<0.01
Vancappel et al. (41)	Maslach Burnout Inventory	Brief COPE	Exhaustion and Active coping	−0.11	<0.01
			Exhaustion and Planning	−0.08	<0.05
			Exhaustion and Instrumental Support	0.05	NS
			Exhaustion and Emotional Support	0.17	<0.01
			Exhaustion and Emotional Expression	0.03	NS
			Exhaustion and Positive Reinterpretation	−0.23	<0.01
			Exhaustion and Acceptance	−0.25	<0.01
			Exhaustion and Denial	0.23	<0.01
			Exhaustion and Blame	0.21	<0.01
			Exhaustion and Humor	−0.16	<0.01
			Exhaustion and Religion	0.03	NS
			Exhaustion and Distraction	−0.20	NS
			Exhaustion and Substance use	0.20	<0.01
			Exhaustion and Behavioral Disengagement	0.31	<0.01
			Depersonalization and Active coping	−0.10	<0.01
			Depersonalization and Planning	−0.08	<0.01
			Depersonalization and Instrumental Support	0.01	NS
			Depersonalization and Emotional Support	0.06	NS
			Depersonalization and Emotional Expression	−0.03	NS
			Depersonalization and Positive Reinterpretation	−0.12	<0.01
			Depersonalization and Acceptance	−0.20	<0.01
			Depersonalization and Denial	0.21	<0.01
			Depersonalization and Blame	0.18	<0.01
			Depersonalization and Humor	−0.01	NS
			Depersonalization and Religion	−0.05	NS
			Depersonalization and Distraction	0.02	NS
			Depersonalization and Substance use	0.17	<0.01
			Depersonalization and Behavioral Disengagement	0.27	<0.01
			Accomplishment and Active coping	0.32	<0.01
			Accomplishment and Planning	0.28	<0.01
			Accomplishment and Instrumental Support	0.16	<0.01
			Accomplishment and Emotional Support	0.08	<0.01
			Accomplishment and Emotional Expression	0.14	<0.01
			Accomplishment and Positive Reinterpretation	0.26	<0.01
			Accomplishment and Acceptance	0.29	<0.01
			Accomplishment and Denial	−0.09	<0.01
			Accomplishment and Blame	−0.03	NS
			Accomplishment and Humor	0.13	<0.01
			Accomplishment and Religion	0.07	<0.05
			Accomplishment and Distraction	0.11	NS
			Accomplishment and Substance use	−0.03	<0.01
			Accomplishment and Behavioral Disengagement	−0.21	<0.01
Michela et al. (42)	Maslach Burnout Inventory	Coping Inventory for Stressful Situations	Emotional Exhaustion and Task-oriented Coping	−0.30	<0.01
			Emotional Exhaustion and Emotion-oriented Coping	0.50	<0.01
			Emotional Exhaustion and Avoidance-Oriented Coping	−0.13	<0.05
			Depersonalization and Task-oriented Coping	−0.32	<0.01
			Depersonalization and Emotion-oriented Coping	0.39	<0.01
			Depersonalization and Avoidance-Oriented Coping	−0.05	NS
			Personal Accomplishment and Task-oriented Coping	0.46	<0.01
			Personal Accomplishment and Emotion-oriented Coping	−0.37	<0.01
			Personal Accomplishment and Avoidance-Oriented Coping	0.14	<0.01
Di Monte et al. (43)	Maslach Burnout Inventory	Coping Inventory for Stressful Situations	Emotional Exhaustion and Emotion-oriented Coping	0.50	<0.001
			Emotional Exhaustion and Task-oriented Coping	−0.25	<0.05
			Emotional Exhaustion and Avoidance-Oriented Coping	0.04	NS
			Depersonalization and Emotion-oriented Coping	0.52	<0.001
			Depersonalization and Task-oriented Coping	−0.22	<0.05
			Depersonalization and Avoidance-Oriented Coping	0.23	<0.05
			Personal Accomplishment and Emotion-oriented Coping	−0.31	<0.001
			Personal Accomplishment and Task-oriented Coping	0.59	<0.001
			Personal Accomplishment and Avoidance-Oriented Coping	0.14	NS
Köse et al. (44)	Maslach Burnout Inventory	Perceived Ability to Cope with Trauma scale	Emotional Exhaustion and Forward Focus	−0.34	<0.01
			Emotional Exhaustion and Trauma Focus	0.96	NS
			Emotional Exhaustion and Flexibility	0.05	NS
			Depersonalization and Forward Focus	−0.22	<0.05
			Depersonalization and Trauma Focus	0.18	<0.05
			Depersonalization and Flexibility	0.15	NS
			Personal Failure and Forward Focus	−0.37	<0.01
			Personal Failure and Trauma Focus	−0.05	NS
			Personal Failure and Flexibility	−0.10	NS
Zhang et al. (45)	Maslach Burnout Inventory (Exhaustion subscale only)	Simplified Coping Style Questionnaire	Exhaustion and Adaptive Coping	OR 0.47 (0.35−0.62)	<0.001
			Exhaustion and Maladaptive Coping	OR 3.28 (2.42−4.45)	<0.001
Liu et al. (46)	Maslach Burnout Inventory	Trait Coping Style Questionnaire	Emotional Exhaustion and Negative coping style	OR 1.99 (1.21−3.26)	0.007
			Depersonalization and Negative coping style	OR 3.47 (2.54−4.73)	<0.001
			Reduced Personal Accomplishments and Negative coping style	OR 1.82 (1.35−2.45)	<0.001
Pang et al. (47)	Copenhagen Burnout Inventory	Brief COPE	Problem-focused coping and Personal-related burnout	−0.07	NS
			Problem-focused coping and Work-related burnout	−0.21	<0.01
			Problem-focused coping and Client-related burnout	−0.26	<0.01
			Emotion-focused coping and Personal-related burnout	0.13	NS
			Emotion-focused coping and Work-related burnout	−0.03	NS
			Emotion-focused coping and Client-related burnout	−0.10	NS
			Avoidance coping and Personal-related burnout	0.38	<0.01
			Avoidance coping and Work-related burnout	0.21	<0.05
			Avoidance coping and Client-related burnout	0.12	NS
Fteropoulli et al. (48)	Copenhagen Burnout Inventory	Brief COPE	Occupational Burnout and Approach coping	-	-
			Occupational Burnout and Support-seeking coping	−0.03	NS (0.269)
			Occupational Burnout and Avoidance coping	0.27	<0.001
AlJhani et al. (49)	Copenhagen Burnout Inventory	Brief COPE	Personal Burnout and Adaptive coping subscales	−0.12	<0.05
			Personal Burnout and Instrumental support	−0.12	<0.05
			Personal Burnout and Emotional support	−0.10	<0.05
			Personal Burnout and Active coping	0.14	<0.01
			Personal Burnout and Planning	−0.05	NS
			Personal Burnout and Positive reframing	−0.10	<0.05
			Personal Burnout and Acceptance	−0.06	NS
			Personal Burnout and Humor	−0.09	NS
			Personal Burnout and Religion	−0.04	NS
			Personal Burnout and Maladaptive coping subscales	−0.04	NS
			Personal Burnout and Self-distraction	−0.08	NS
			Personal Burnout and Denial	−0.06	NS
			Personal Burnout and Self-blaming	0.07	NS
			Personal Burnout and Behavioral disengagement	0.06	NS
			Personal Burnout and Venting	−0.05	NS
			Personal Burnout and Substance use	−0.09	NS
			Work-related Burnout and Adaptive coping subscales	−0.11	<0.05
			Work-related Burnout and Instrumental support	−0.09	NS
			Work-related Burnout and Emotional support	−0.11	<0.05
			Work-related Burnout and Active coping	−0.20	<0.01
			Work-related Burnout and Planning	−0.06	NS
			Work-related Burnout and Positive reframing	−0.10	<0.05
			Work-related Burnout and Acceptance	−0.07	NS
			Work-related Burnout and Humor	−0.04	NS
			Work-related Burnout and Religion	−0.05	NS
			Work-related Burnout and Maladaptive coping subscales	−0.01	NS
			Work-related Burnout and elf-distraction	−0.11	<0.05
			Work-related Burnout and Denial	-.05	NS
			Work-related Burnout and Self-blaming	0.14	<0.01
			Work-related Burnout and Behavioral disengagement	0.06	NS
			Work-related Burnout and Venting	−0.03	NS
			Work-related Burnout and Substance use	−0.04	NS
			Client-related Burnout and Adaptive coping subscales	−0.16	<0.01
			Client-related Burnout and Instrumental support	−0.09	NS
			Client-related Burnout and Emotional support	−0.07	NS
			Client-related Burnout and Active coping	−0.24	<0.01
			Client-related Burnout and Planning	−0.06	NS
			Client-related Burnout and Positive reframing	−0.17	<0.01
			Client-related Burnout and Acceptance	−0.13	<0.05
			Client-related Burnout and Humor	−0.04	NS
			Client-related Burnout and Religion	−0.17	NS
			Client-related Burnout and Maladaptive coping subscales	0.02	NS
			Client-related Burnout and Self-distraction	−0.13	<0.05
			Client-related Burnout and Denial	−0.01	NS
			Client-related Burnout and Self-blaming	0.18	<0.01
			Client-related Burnout and Behavioral disengagement	0.06	NS
			Client-related Burnout and Venting	−0.04	NS
			Client-related Burnout and Substance use	−0.01	NS
Fonseca et al. (50)	Oldenburg Burnout Inventory	Brief COPE	Cognitive reappraisal and Burnout	−0.10	NS (0.305)
			Expressive suppression and Burnout	0.45	<0.001
			Problem focused coping and Burnout	−0.17	NS (0.096)
			Emotion-focused coping and Burnout	−0.14	NS (0.202)
			Dysfunctional coping and Burnout	0.52	<0.001
Finuf et al. (51)	Oldenburg Burnout Inventory	Cybernetic Coping Scale	Symptom Reduction and Disengagement	−0.15	NS
			Devaluation strategies and Disengagement	−0.31	<0.05
			Avoidance strategies and Disengagement	0.71	<0.001
			Change Situation and Disengagement	−0.11	NS
			Accommodation strategies and Disengagement	0.14	NS
			Symptom Reduction and Exhaustion	−0.19	NS
			Devaluation strategies and Exhaustion	−0.24	NS
			Avoidance strategies and Exhaustion	0.12	NS
			Change Situation and Exhaustion	−0.13	NS
			Accommodation strategies and Exhaustion	0.28	NS
Sumner et al. (52)	Bergen Burnout Inventory	Brief Resilient Coping Scale	Burnout and resilient coping	−0.01	NS
Miller et al. (53)	Professional Quality of Life	Brief COPE	Burnout and behavioral disengagement	3.17	<0.001
			Burnout and humor	1.56	<0.01

A quality evaluation was performed on the included studies, using the NOS (38); all the included studies were at least at a good quality level on the scale (six points or higher) (Table 3).

**TABLE 3:** Quality assessment of included studies through Newcastle-Ottawa scale.

References	Selection	Comparability	Outcome	Total score
Lungulescu et al. (39)	4	2	2	8
Crescenzo et al. (40)	3	1	2	6
Vancappel et al. (41)	3	1	2	6
Michela et al. (42)	4	1	2	7
Di Monte et al. (43)	3	2	2	7
Köse et al. (44)	2	2	2	6
Zhang et al. (45)	4	2	2	8
Liu et al. (46)	3	2	2	7
Pang et al. (47)	4	2	2	8
Fteropoulli et al. (48)	4	2	2	8
AlJhani et al. (49)	4	2	2	8
Fonseca et al. (50)	3	1	2	6
Finuf et al. (51)	3	1	2	6
Sumner et al. (52)	3	2	2	7
Miller et al. (53)	4	1	2	7

Results are presented in different sections based on burnout model, first unidimensional and then multidimensional. For studies using a multidimensional burnout model, results are divided based on coping style (task-, emotion- or avoidance-oriented coping) when investigated, or adaptive/maladaptive coping style. For each paragraph, protective factors (negative correlation) are reported first, and predictive factors (positive correlation) are reported after.

### 3.1. Unidimensional burnout models and coping

Concerning protective factors, Lungulescu et al. (39) highlighted that that active approach coping and positive interpretation were protective factors for burnout (*p* < 0.001). Pang et al. (47) highlighted problem-focused coping as a protective factor for work- and client-related burnout (*p* < 0.01); AlJhani et al. (49) found that active coping and self-distraction were protective for work- and client-related burnout (*p* < 0.01), and positive reframing was a protective factor for personal (*p* < 0.05), work- (*p* < 0.05) and client-related burnout (*p* < 0.01); furthermore, this study reported that adaptive coping subscales were negatively correlated to personal, work- and client-related burnout (*p* < 0.05, *p* < 0.05, and *p* < 0.01, respectively).

In regards to burnout predictive factors, Lungulescu et al. (39) highlighted that expressing emotions, as well as both mental and behavioral deactivation, are predictive factors of burnout (*p* < 0.001). Vancappel et al. (41) highlighted that emotional expression was predictive of burnout (*p* < 0.01). Pang et al. (47) reported avoidance coping as a predictor of personal- (*p* < 0.01) and work-related burnout (*p* < 0.05). Fteropoulli et al. (48) reported avoidance coping as a predictive factor for occupational burnout (*p* < 0.001). AlJhani et al. (49) found that active coping was predictive of burnout (*p* < 0.01). Fonseca et al. (50) reported that dysfunctional coping was predictive of burnout (*p* < 0.001). Miller et al. (53) highlighted behavioral disengagement as predictive of burnout (*p* < 0.001).

### 3.2. Burnout dimensions and task-oriented coping

Four of the included studies highlighted a statistically significant correlation between task-oriented coping and burnout dimensions (40–43).

Vancappel et al. (41), Crescenzo et al. (40), Di Trani et al. (42), and Di Monte et al. (43), highlighted that task-oriented coping was a protective factor for exhaustion dimension (*p* < 0.01, *p* < 0.05, *p* < 0.01, and *p* < 0.05, respectively), while only Vancappel et al. (41), Di Trani et al. (42) and Di Monte et al. (43) found a statistically significant correlation between depersonalization and task-oriented coping (*p* < 0.01, *p* < 0.01, and *p* < 0.05, respectively).

These four studies highlighted that task-oriented coping was a predictive factor of personal accomplishment (*p* < 0.01, *p* < 0.01, *p* < 0.01, and *p* < 0.001, respectively) (40–43).

### 3.3. Burnout dimensions and emotion-oriented coping

Four of the included studies reported a statistically significant correlation between emotion-oriented coping and burnout dimensions (40–43).

Two (Crescenzo et al and Vancappel et al) found that emotion-oriented coping was a protective factor for emotional exhaustion and depersonalization (*p* < 0.01 for both) (40, 41). The other two studies (Di Trani et al and Di Monte et al) found emotion-oriented coping to be negatively correlated with personal accomplishment (*p* < 0.01 and *p* < 0.001, respectively) (42, 43).

Crescenzo et al. (40) and Vancappel et al. (41) highlighted emotion-oriented coping as a predictive factor for personal accomplishment (*p* < 0.01 for both). The other two studies (Di Trani et al and Di Monte et al) found a positive correlation with emotional exhaustion and depersonalization (*p* < 0.01 and *p* < 0.001, respectively) (42, 43).

### 3.4. Burnout dimensions and avoidance-oriented coping

Five studies investigated the correlation between avoidance-oriented coping and burnout (40–43, 51).

In regards to protective factors, Crescenzo et al. (40) found that avoidance strategies were negatively correlated with personal accomplishment (*p* < 0.01), while Di Trani et al. (42) highlighted that avoidance-oriented coping was a protective factor for emotional exhaustion (*p* < 0.05). Vancappel et al. (41) reported that behavioral disengagement was predictive of accomplishment (*p* < 0.01).

Concerning predictive factors, Crescenzo et al. (40) found that avoidance strategies positively correlated with emotional exhaustion and depersonalization (*p* < 0.01). Di Monte et al. (43) also reported a positive correlation between depersonalization and avoidance-oriented coping (*p* < 0.05). Di Trani et al. (42) highlighted that avoidance-oriented coping correlated and positively with personal accomplishment (*p* < 0.01). Vancappel et al. (41) also reported behavioral disengagement as predictive of emotional exhaustion and depersonalization (*p* < 0.01). Finuf et al. (51) highlighted avoidance strategies as predictive of disengagement (*p* < 0.05).

### 3.5. Burnout dimensions and adaptive/maladaptive coping

Two studies investigated the correlation between burnout and adaptive or maladaptive coping strategies (45, 46).

Zhang et al. (45) reported that individuals using adaptive coping were less at risk of developing exhaustion [OR 0.47, CI (0.35–0.62), *p* < 0.001], while individuals using maladaptive coping strategies were more at risk of developing exhaustion [OR 3.28, CI (2.42–4.45), *p* < 0.001]. Liu et al. (46) highlighted that a negative coping style posed participant at risk of developing emotional exhaustion [OR 1.99, CI (1.21–3.26), *p* = 0.007], depersonalization [OR 3.47, CI (2.54–4.73), *p* < 0.001], and reduced personal accomplishment [OR 1.82, CI (1.35–2.45), *p* < 0.001].

## 4. Discussion

The systematic analysis of studies on the coping strategies adopted by workers during the COVID-19 pandemic and the levels of burnout associated with them, allowed us to observe some significant associations. All studies investigating task-oriented coping strategies observed that they were associated with low levels of burnout, so seeming an effective way of reducing it. Task-oriented coping could act as a protective factor toward emotional exhaustion and depersonalization, as well as a predictor of personal accomplishment. Task-oriented coping allows the individual to deal with the problem directly, facing the issue in a healthy manner that contributes to mitigate the stressor and reduce or prevent burnout. This is coherent with scientific literature, as task-oriented coping has been showcased to be an effective approach to reduce stress during the COVID-19 pandemic (54). Interestingly, task-oriented coping has been shown to be more commonly adopted in the general population during the earlier pandemic stages, although its effectiveness did not decrease in time (55). The effectiveness of a coping style focused on problem solving appears even more important when considering that the three of the studies included in this review which investigated this relationship were performed on healthcare workers and one on psychologists. The ability of healthcare workers to deal with burnout by managing the problem or task acting as a stressor may play a fundamental role in healthy coping and in reducing burnout levels, allowing healthcare workers to keep caring for others during highly stressful times, such as the COVID-19 pandemic.

This review found mixed evidence of the relationship between emotion-oriented coping and burnout. Considering the four studies correlating burnout dimensions and emotional-oriented coping, two highlighted that this coping style was effective in preventing emotional exhaustion and depersonalization, and predicting personal accomplishment, while the other two showcased the opposite. The samples of these four studies do not differ much; the first two considered psychologists and healthcare workers, respectively, the other two considered healthcare workers. Mean ages were also similar (around 40 years for the first three studies, and higher for the fourth at 55.13 years old). A higher prevalence of the female gender (83%) has been reported in the studies that considered emotional-oriented coping as effective in preventing burnout, if compared with a lower female percentage (65.2% and 62.7%) in the other studies. Literature indicates that emotion-oriented coping is a strategy more frequently adopted by women (56, 57), and with more favorable outcomes than those observed in men (58). Subsequent studies may clarify the gender differences between coping strategies and the relative effectiveness of the different strategies in the two sexes. To date, we can conclude that emotional-oriented coping is not always effective in burnout prevention.

Avoidance-oriented coping style was associated with burnout and probably acted as a predictive factor for emotional exhaustion and depersonalization, reducing personal accomplishment. As reported in previous studies, avoidant behavior seems to be ineffective in preventing burnout (59), and can worsen it when the stressor cannot be removed from the working life (60, 61).

Consistently with the findings from this literature review, adaptive coping has been correlated with lower levels of burnout (62, 63). Also the association between psychological distress and maladaptive coping has been reported in previous studies (64).

This study had some strength and limitations. The systematic design allowed for a structured search through scientific literature regarding the topic presented. However, the interpretation of the studies reported above must be cautious, because all the studies found were of the cross-sectional type and this prevents inferring about the causality of the reported phenomena. Consequently, the reported associations should not be interpreted as conclusive evidence of causality. If, in fact, it is reasonable to believe that the state of burnout is a consequence of strategies to contrast stress implemented by the worker in the past, the cross-sectional nature of the studies did not allow us to exclude the inverse hypothesis, that is, that it was the burnout condition to induce a particular coping strategy. Furthermore, during the COVID-19 pandemic workers reported higher stress and burnout levels due to remote working and factors associated with it, and this may have acted as a confounding factor in the review (6).

In conclusion, task-oriented coping and adaptive coping appeared to be effective ways to deal with burnout during the COVID-19 pandemic, while emotion-oriented coping has different outcomes that may depend on gender. Avoidance-oriented coping and maladaptive coping are often associated with high levels of burnout and could be predictors.

Coping strategies are mostly used restoratively; it would be interesting to implement coping as a tool to prevent burnout. Burnout prevention through coping strategies has been highlighted by existing scientific literature as an essential tool, which needs further investigation and improvement (65). This systematic review highlights that incorrect coping styles are associated with burnout and can enhance it (40–43), but further research is needed to investigate the effect of different coping styles to reduce burnout incidence. It is essential to develop and implement effective coping strategies as a preventive measure to ensure the mental and physical wellbeing of workers. Furthermore, workplace information and prevention programs may be needed to instruct workers on the best coping mechanisms to enact in order to effectively prevent burnout; this review offers a starting point to identify and improve effective coping strategies to workers at risk for burnout. It is essential to instruct employee, especially those working in a stressful working environment, about the best coping strategies that can help mitigate their psychological risks and improve their mental health.

In line with the results of this systematic review, further research is needed to investigate how different coping styles affect burnout in workers, and especially how coping strategies can affect the work climate and result in a stressful work environment.

Effective coping strategies have been highlighted in this review to be instrumental to mitigate burnout, but it is essential to investigate the role of coping styles and strategies in the prevention of mental distress, to be able to act before the stressors become detrimental for the workers, and enact effective preventive measures for mental health in workers.

## Data availability statement

The original contributions presented in this study are included in this article/supplementary material, further inquiries can be directed to the corresponding author.

## Author contributions

IB: conceptualization. MG: methodology. MR and IB: investigation and writing—original draft. IB, MG, PS, UM, and NM: writing—review and editing. IB, PS, and UM: supervision. All authors contributed to the article and approved the submitted version.
